# The Interface and Mechanical Properties of a CVD Single Crystal Diamond Produced by Multilayered Nitrogen Doping Epitaxial Growth

**DOI:** 10.3390/ma12152492

**Published:** 2019-08-06

**Authors:** Yun Zhao, Chengming Li, Jinlong Liu, Kang An, Xiongbo Yan, Lifu Hei, Liangxian Chen, Junjun Wei, Fanxiu Lu

**Affiliations:** Institute for Advanced Materials and Technology, University of Science and Technology Beijing, Beijing 100083, China

**Keywords:** multilayers, nitrogen doping, single crystal diamond, X-ray topography, dislocation, mechanical property

## Abstract

In the present investigation, a nitrogen-doped multilayer homoepitaxial single crystal diamond is synthesized on a high-pressure high temperature (HPHT) Ib-type diamond substrate using the microwave plasma chemical vapor deposition (MPCVD) method. When 0.15 sccm of nitrogen was added in the gas phase, the growth rate of the doped layer was about 1.7 times that of the buffer layer, and large conical and pyramidal features are formed on the surface of the sample. Raman mapping and photoluminescence imaging of the polished cross sectional slice shows a broadband emission, with a characteristic zero phonon line (ZPL) at 575 nm in the doped layers, and large compressive stress was formed in the nitrogen-doped layers. X-ray topography shows that the defects at the interface can induce dislocation. The pyramid feature is formed at the defect, and more nitrogen-related defects are formed in the pyramid region. Thin nitrogen-doped multilayers were successfully prepared, and the thickness of the nitrogen-doped and buffer layers was about 650 nm each. The indentation measurements reveal that the thin nitrogen-doped multilayers are ultra-tough (at least ~22 MPa m^1/2^), compared to the Ib-type HPHT seed substrate (~8 MPa m^1/2^) and the unintentionally doped chemical vapor deposition (CVD) single crystal diamond (~14 MPa m^1/2^).

## 1. Introduction

It is well known that single crystal diamonds can be synthesized at a very high growth rate by microwave plasma chemical vapor deposition (MPCVD) [1,2]. These diamonds have already shown excellent mechanical properties, which has opened up new prospects for certain novel applications, such as diamond anvils [3]. High fracture toughness (6–18 MPa m^1/2^), and an abnormally high hardness (~160 GPa) could be obtained by a high pressure and high temperature (HPHT) post growth treatment. It has been shown that when such a single crystal diamond is heat-treated, a combination of unusual mechanical properties can be obtained [4,5], thus introducing a novel method to harden the chemical vapor deposition (CVD) single crystal diamonds. Controlling the microstructure of the single crystal diamond can further enhance its strength and hardness [6,7]. The multilayered method has been successfully used to improve the hardness and toughness of ceramic coatings [8,9]. The mechanisms may possibly be due to the blocking of the dislocation movement between the layers. The key of the toughening is the creation of complex coherent boundaries at the interfaces, whereby more energy is consumed during crack initiation and propagation [8]. The multilayered method may provide a new way to expand the application of the single crystal diamonds by optimizing their structures.

Nitrogen is the most widely studied impurity in diamonds [10,11]. Natural and HPHT diamonds that are rich in nitrogen appear yellow [12]. Modern MPCVD technology makes it possible to obtain epitaxial diamond layers of very high crystalline perfection with controlled impurity content [13]. A CVD diamond can be doped with nitrogen impurities from the gas phase. Nitrogen in a CVD diamond usually exists in the form of substitutional nitrogen and nitrogen-vacancy centers (NV) [14]. The negatively charged NV defects (NV^−^) in diamonds have significant optical and electron spin properties, which can be used in quantum information processing [15,16] and highly sensitive magnetic force measurement [17]. Doping with nitrogen reduces the energy barrier for the release of thermal electrons in solar energy converters [18]. Some applications require high nitrogen-doping concentrations at the near-surface nanoscale thin layer of the diamond [19]. Hence, research is needed on the influence of nitrogen doping on CVD diamond growth [20,21,22,23,24] to improve their properties.

Diamond’s high Young’s modulus and hardness make it an attractive material for various applications. However, attempting to deform diamonds usually results in brittle fracture [25,26,27,28]. Design of tough material can be realized by inducing compressive stress [29] or optimizing multilayered structure [8]. The main purpose of this work is to improve the toughness of the single crystal diamonds by epitaxial growth of thin nitrogen-doped multilayers.

## 2. Materials and Methods 

Multilayered epitaxial layers doped with nitrogen were grown in a custom 2.45 GHz quartz bell jar type CVD reactor. In the experiments, we used Ib-type HPHT diamond substrates with an orientation of (100) and size of 4.0 × 4.0 × 1.0 mm^3^ with a nitrogen content <60 ppm from SINO CRYSTAL (Zhengzhou, China). Hydrogen (5 N) and methane (5 N) gases were used as the process gas. The substrates were mechanically polished to a surface roughness (Ra) of 3 nm. After polishing, the seed substrates were acid cleaned with a mixture of sulfuric and nitric acids (The ratio of mixture is 5:1). Then the substrates were etched with an O_2_/H_2_ (2/98) plasma treatment before the growth to remove the defects in the near-surface region introduced by polishing. The incorporation of nitrogen impurities during CVD diamond growth is dependent on the substrate orientation, and can be affected by the misorientation angle. Therefore, in order to eliminate the effect of the misorientation angle on different substrates, several nitrogen-doped layers were designed to grow on one sample. A growth power of 2500 W, the reactor was maintained at a pressure of 25 kPa and a temperature of 1000 °C. The growth conditions were changed from layer to layer. The buffer layers were grown in a gas mixture of CH_4_ + H_2_ (CH_4_/H_2_ = 5%) with no intentional addition of N_2_. Nitrogen incorporation dependent on the nitrogen flow was investigated using sample 1. To grow nitrogen-doped multilayers for sample 1, this mixture was rapidly changed to a gas mixture containing nitrogen CH_4_ + H_2_ + N_2_ (CH_4_/H_2_ = 5%) using a gas switch, and the gas flow rate was 300 standard cubic centimeter per minute (sccm) for H_2_, N_2_ = 0.03, 0.06, 0.09, 0.12, and 0.15 sccm (The ratios to CH_4_ were 0.2%, 0.4%, 0.6%, 0.8% and 1.0%), and the growth duration for each layer was 2 h. The total thickness of the CVD layer was about 680 μm. The response time of the switch was not more than 10 s. The residence time t required for changing the gas composition in the chamber as defined by the equation [20]: t = (V × P)/(F × P_atm_), where V is the chamber volume (~1.4 L), and P is the pressure (~15 kPa), and F is the total gas flow injected in the chamber (~315 sccm), and P_atm_ is the atmospheric pressure (~100 kPa) and the residence time t ≈ 35 s. So, the total time from closing the switch to the disappearance of nitrogen in the chamber is response time plus residence time (about 45 s).

In order to obtain thinner nitrogen-doped layers, sample 2 was grown under a growth condition, which included 2700 W of microwave plasma power, 17 kPa of pressure, a temperature of 880 °C and a nitrogen flow of 0.09 sccm. CH_4_ and H_2_ in a ratio of 5/95 were used. For sample 2, the single layer growth time of nitrogen-doped layer was reduced to 5 min. Each nitrogen-doped and buffer layer was about 650 nm thick. The total thickness of the CVD layer was about 35 μm. To assess whether thin nitrogen-doped multilayers can improve the mechanical properties of a single crystal diamond, another sample, labeled 3, was prepared using the same growth parameters as sample 2 but without nitrogen addition. Table 1 shows the growth parameters of different samples. Sample 1 was a multilayer structure consisting of different nitrogen content. Sample 2 was a multilayer structure consisting of the same nitrogen content. Sample 3 was a single layer structure without nitrogen addition.

Morphology of the surface and the cross section were investigated using an optical microscope. The surface morphology of the indentation was observed using a scanning electron microscope (SEM) (FEI, QUANTA FEG 250, Hillsboro, OR, USA). The cross section was obtained by laser-cutting and polishing. Raman mapping of the cross section was studied with a laser-scanning Raman microscope (Nanophoton Corporation, Osaka, Japan) under an optical excitation wavelength of 532 nm, and the spatial resolution was about 260 nm. All Raman spectra were recorded at room temperature, and the spectral resolution is 0.4 cm^−1^. The ultraviolet (UV) photoluminescence (PL) of the samples was characterized using the DiamondView^TM^ equipment (De Beers Group company, London, UK), which exposes samples to the above-band gap UV radiation to excite the surface luminescence and can provide the information about spatial variations in the intensity of the luminescence emanating from the point defects. The extended defects propagating in the CVD layers were characterized with synchrotron radiation X-ray topography (XRT) (Chinese Academy of Sciences, Beijing, China). The following were the main parameters of XRT: The distance between the sample and the film was 5.5 cm, and the sample was about 43 m from the X-ray source. The operating parameters of the storage ring were 2.5 GeV and 250 mA. Secondary ion mass spectroscopy (SIMS) (Cameca IMS 4f, Gennevilliers, France) was used to measure the nitrogen concentration in the nitrogen-doped multilayer sample. A double-focusing magnetic secondary ion mass spectrometer (Cameca IMS 4f, Gennevilliers, France) was used for the depth profiling, and an Alpha-Step 500 Surface Profiler (Cameca IMS 4f, Gennevilliers, France) was used to measure the depths of the etching craters for the analysis depth scale. Sputtering was performed using Cs^+^ ions with an energy of 14.5 keV. The SIMS nitrogen measurement was quantitatively calibrated using a single crystal diamond with a nitrogen concentration of 5.93 × 10^17^ cm^−3^. A micro-Vickers hardness tester (Shanghai Lianer Testing Equipment Co., Ltd., Shanghai, China) was used to evaluate the Vickers hardness (Hv) and the fracture toughness (Kc), which were measured with a load of 9.8 N and a holding time of 10 s.

## 3. Results and Discussion

### 3.1. Nitrogen-Doping Dependence on the Nitrogen Flow

**Figure 1:**
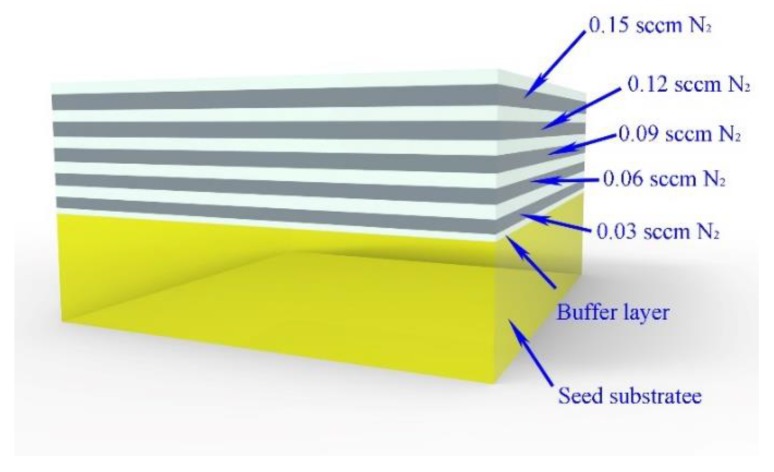
Schematic of the chemical vapor deposition (CVD) layers of sample 1 with various amounts of added N_2_. The white and light gray layers correspond to the buffer and the nitrogen-doped layers, respectively. The yellow part corresponds to the Ib-type high-pressure high temperature (HPHT) seed substrate.

Figure 1 shows the schematic of the CVD layers of sample 1 with a variable amount of added N_2_.

**Figure 2:**
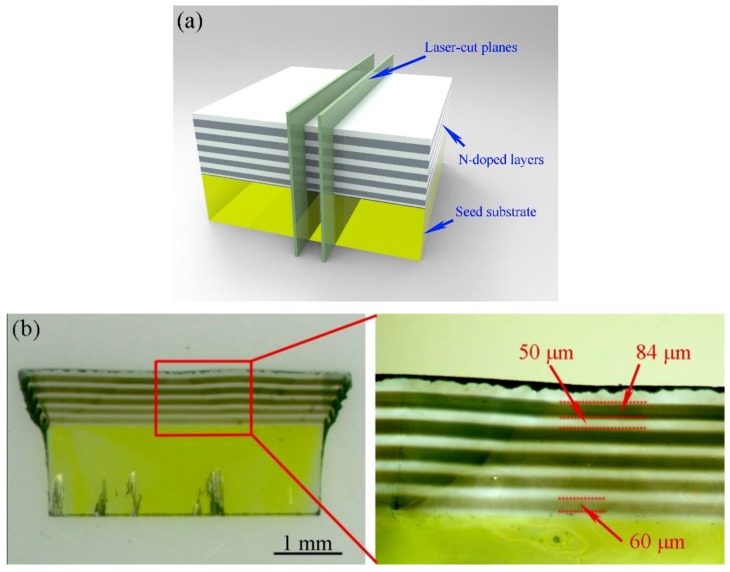
(**a**) Schematic of laser cutting of the nitrogen-doped multilayers grown on the HPHT seed substrate. The two cutting planes perpendicular to the surface are shown in light green. (**b**) Optical image of the 400-μm-thick cross section slice obtained after laser-cutting and polishing of the lateral faces.

To observe and analyze the multilayered structure, a 400-μm-thick cross section slice was laser cut along the planes perpendicular to the sample’s surface, as seen in Figure 2a,b, showing a slice on which the lateral faces are polished. We can clearly distinguish the alternating growth of the nitrogen-doped layers (dark stripes) and the buffer layers (light stripes). As the nitrogen content increases, the thickness of the nitrogen-doped layer increases, and the color deepens. The influence of this element on the growth rate has been widely reported [21], and it is known that the growth rate increases rapidly when nitrogen is added during growth. We observed that a low nitrogen concentration in the gas phase is sufficient to dramatically increase the growth rate. When 0.15 sccm of nitrogen was added in the gas phase, the thickness of the doped layer was 84 μm, whilst the buffer layer was only 50 μm, so the growth rate of the doped layer was about 1.7 times that of the buffer layer. When 0.03 sccm of nitrogen was added in the gas phase, the thickness of the doped layer was 60 μm, which was also thicker than the buffer layer. As expected, the growth rate was strongly enhanced by the presence of N_2_.

**Figure 3:**
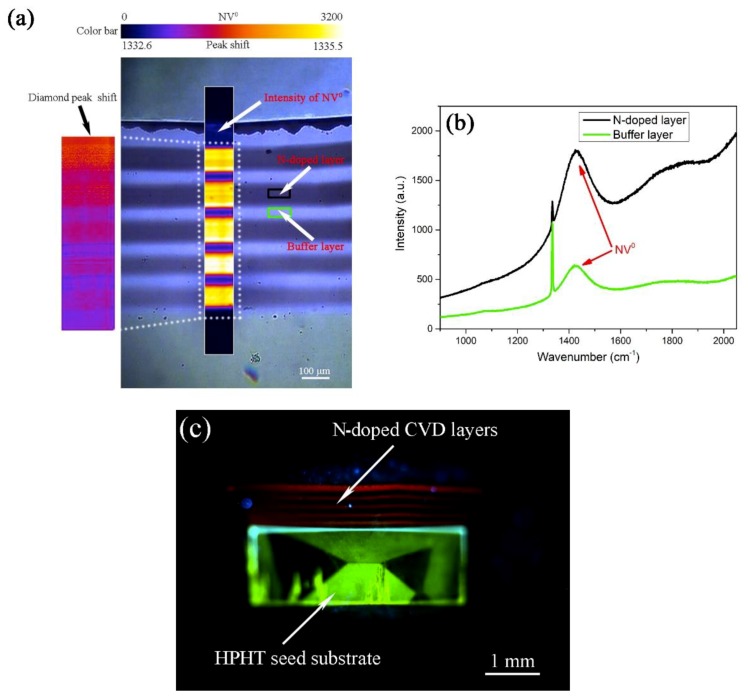
(**a**) Laser-scanning Raman images show the Raman mapping of the diamond peak shift (left inset) and the fluorescence intensity of the NV^0^ defect of the cross section slice (white dotted line area). Correspondingly, the diamond peak shift (1332.6–1335.5 cm^−1^) and the fluorescence intensity of NV^0^ (0–3200 counts) are shown in the same color bar on the top. (**b**) Raman spectra recorded at room temperature for the 0.12 sccm nitrogen-doped layer and the buffer layer, respectively. (**c**) UV-excited photoluminescence image of the polished cross sectional slice of the nitrogen-doped multilayer CVD diamond grown on the Ib-type HPHT substrate.

Raman mapping of the cross section slice is shown in Figure 3a. The diamond peak shift (1332.6–1335.5 cm^−1^) and the fluorescence intensity of NV^0^ (0–3200 counts) are shown in the same color bar on the top. High fluorescence signal can be seen in the nitrogen-doped layers. In these regions, the Raman spectrum exhibits a broad peak centered at 1420 cm^−1^, which can be associated with the broadband emission (with zero phonon-line at 575 nm) typical of the nitrogen-vacancy NV^0^ defect [24]. The left inset in Figure 3a shows Raman mapping of the diamond peak shift in the white dotted line area. Raman mapping shows an obvious shift in the diamond peaks toward the direction of the higher wavenumber, which indicates that huge compressive stress exists in the nitrogen-doped layers [30]. The intensity of the 1420 cm^−1^ peak in the N-doped layer (0.12 sccm) is roughly 3 times that of the same peak in the buffer layer, and that means about a triple density of NV^0^ defect in the N-doped layer with respect to the buffer one, as shown in Figure 3b [23]. Figure 3c is a photoluminescence image of the cross sectional slice of the sample 1, which is measured by DiamondView. The orange-red luminescence is predominantly from the NV^0^ defect, and green luminescence emanates from the Ib-type HPHT-grown seed substrate [31].

**Figure 4:**
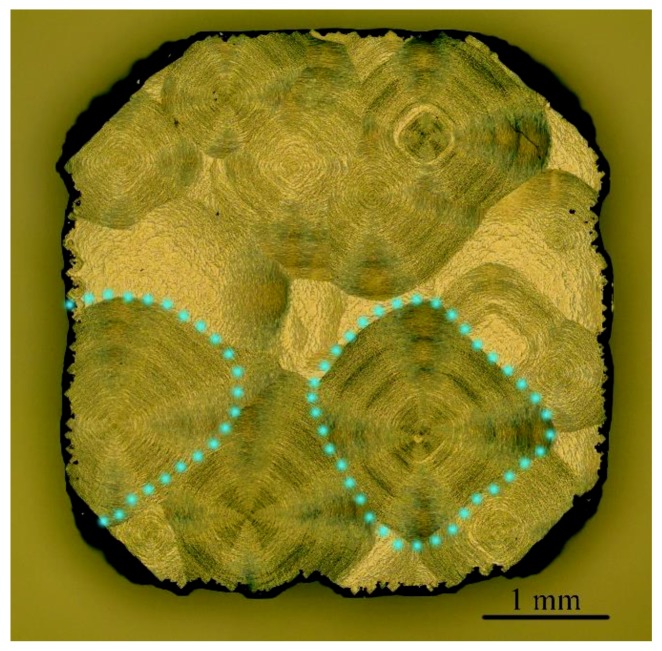
Optical microscope image of the surface morphology of sample 1 prepared with intentional addition of N_2_. The blue dotted lines mark the conical and pyramidal features on the surface.

Figure 4 shows the optical microscope image of the surface morphology of the sample 1 after all the deposition process. Classical step bunching leading to the formation of the macro-step can be observed. Large conical and pyramidal features are formed on the surface of the sample, which are marked with blue dotted lines. In earlier works, when the nitrogen concentration in the gas phase was high, these pyramidal features and the corners were more rounded [32]. This evolution of the shape was due to the decrease of the step velocity limited by surface diffusion, which showed that the addition of nitrogen was equivalent to an increase of carbon supersaturation. In these conditions, the growth mode changed from step flow to bi-dimensional nucleation [33].

**Figure 5:**
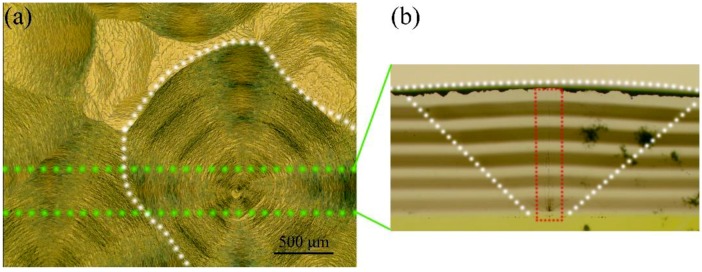

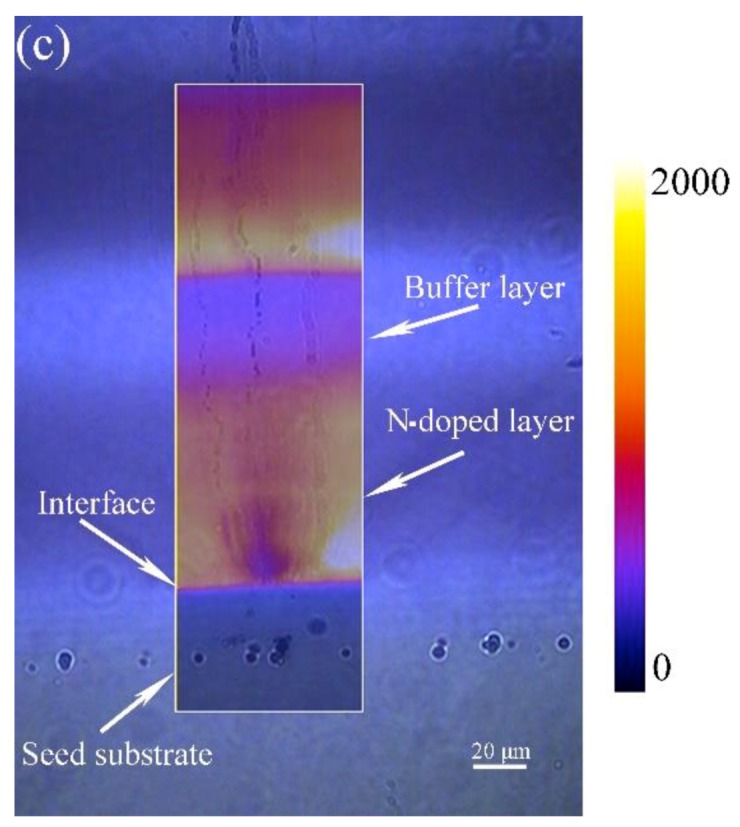
(**a**,**b**) Optical images showing the surface morphology and the cross section of the pyramidal feature, respectively. (**c**) Raman image of the defect formed at the interface was recorded by measuring the signal intensity at 1420 cm^−1^ (corresponding to the emission from the NV^0^ defect).

Figure 5a shows the surface morphology of the pyramidal feature, which reflects the high density of macro-steps that is commonly observed in the presence of a higher amount of nitrogen [32,33], since nitrogen impurities are more easily formed into macro-steps [34]. Figure 5b shows the cross section of the pyramidal feature, which is marked by white dotted lines. The pyramid area is darker brown than the other areas, which indicates that more nitrogen-related impurities are formed in the pyramid area. The red dotted line marks the black defect formed at the interface. Raman image was recorded for the black defect in Figure 5c, which shows that the black defect has a lower amount of nitrogen impurities. We speculate that the black defect might be the non-diamond phase.

**Figure 6:**
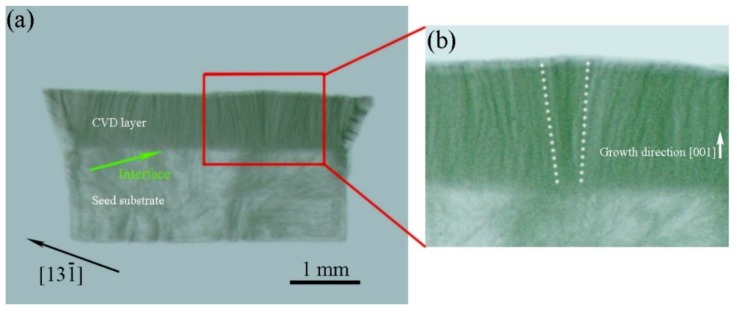

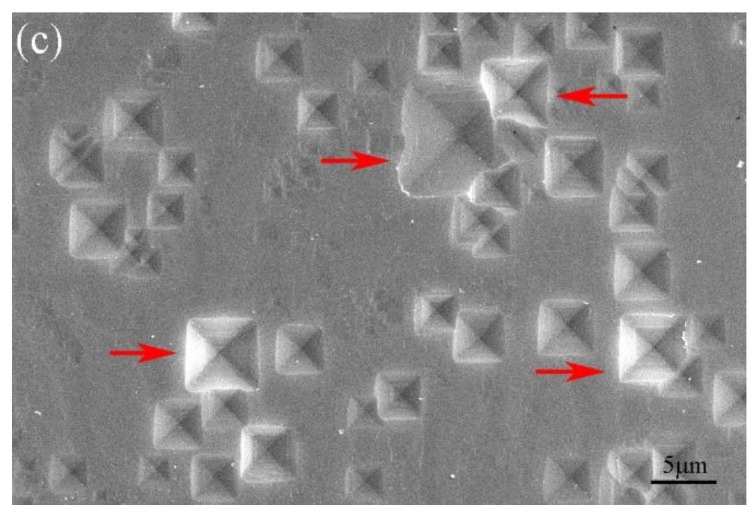
(**a**) XRT image of the (100)-cut slice of the sample 1 showing the interface between the HPHT seed substrate and the CVD layer, which is indicated by the green arrow. (**b**) Magnified XRT image of the pyramidal feature. The image shows that a number of dislocations are formed at the defect, and some of the dislocations are bent along the direction of the step flow. (**c**) Optical microscope image of surface morphology of the HPHT seed substrate after plasma etching.

A synchrotron radiation XRT image of the slice is shown in Figure 6a. The interface between the seed substrate and the CVD layer is indicated by the green arrows. In this image, some of the dislocations originated from the initial seed substrate. However, most of the dislocations are induced by the interface and are generated during growth. Figure 6b shows the magnified XRT image of the pyramidal feature. A number of dislocations formed at the defect, and some of the dislocations in the pyramidal area are bent along the direction of the step flow. Almost all of the dislocations deviate from the direction of propagation, which is typically [001], and these dislocations are not perpendicular to the surface. Step-flow growth can deflect dislocations away from the primary growth direction in the homoepitaxial growth of diamond on (001) seed substrate [35]. In order to remove the polishing-induced layer of damage on the seed substrate, the HPHT seed substrate was etched in a CVD reaction chamber. Figure 6c shows the surface morphology of HPHT seed substrate after plasma etching. The seed substrate had some large etch pits indicated by red arrows after etching. The surface of the seed substrate was removed by 2 microns, and the surface roughness (Ra) was about 70 nm. Larger surface roughness may result in a non-diamond phase and inducing more dislocations.

The presence of defects on the substrate has consequences in terms of morphology, these defects can induce numerous dislocations that propagate through the layers [36]. Adopting appropriate process parameters can partially reduce these defects and their influence on layer growth. The growth of the high-quality CVD single crystal diamonds also requires careful substrate selection.

### 3.2. Growth of Thin Nitrogen-Doped Layers

**Figure 7:**
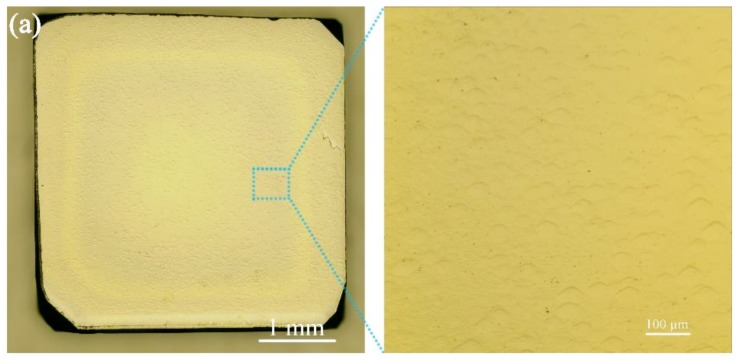

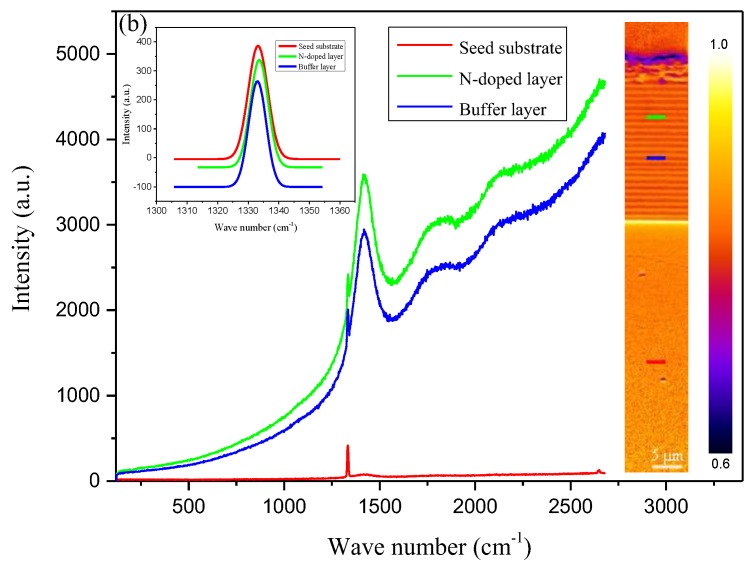
(**a**) Surface morphology of sample 2. (**b**) Raman scan of the cross section of sample 2 showing the presence of alternating thin nitrogen-doped and buffer layers. The inset shows the Raman spectra of the first-order phonon peak of diamond in different positions.

There is a coupled effect of nitrogen addition and surface temperature on the morphology of CVD single crystal diamond [32]. This point shows that it should design the nitrogen incorporation and the growth rates by choosing an appropriate growth temperature. In order to obtain thinner nitrogen-doped layers, we should lower the growth temperature and nitrogen concentration. Another structure (sample 2) was grown by intentional addition of 0.09 sccm of N_2_ and shorter growth duration (5 min) at 880 °C. Figure 7a shows the surface morphology of sample 2 which is smooth compared with sample 1. The thickness of the resulting nitrogen-doped layer is about 650 nm. This amount of nitrogen was sufficient to generate a detectable intensity of the NV^0^ defect in the vertical growth sector in Figure 7b. We can obtain the biaxial stress state of the thin nitrogen-doped layer and the buffer layer according to the following equation [37]: P = −0.61 GPa/cm^−1^. The obtained Raman spectra indicate shifts of the diamond peaks in the direction of higher wavenumbers. In the nitrogen-doped layer, the diamond peak shifts to 1333.6 cm^−1^, and the difference from the diamond peak without stress 1332.5 cm^−1^ is 1.1 cm^−1^, corresponding to a stress level of 0.67 GPa. In the buffer layer, the diamond peak shifts to 1333.0 cm^−1^, and the difference from the diamond peak without stress 1332.5 cm^−1^ is 0.5 cm^−1^, corresponding to a stress level of 0.31 GPa. The compressive stress of the nitrogen-doped layer is more than twice that of the buffer layer, as shown in Figure 7b.

**Figure 8:**
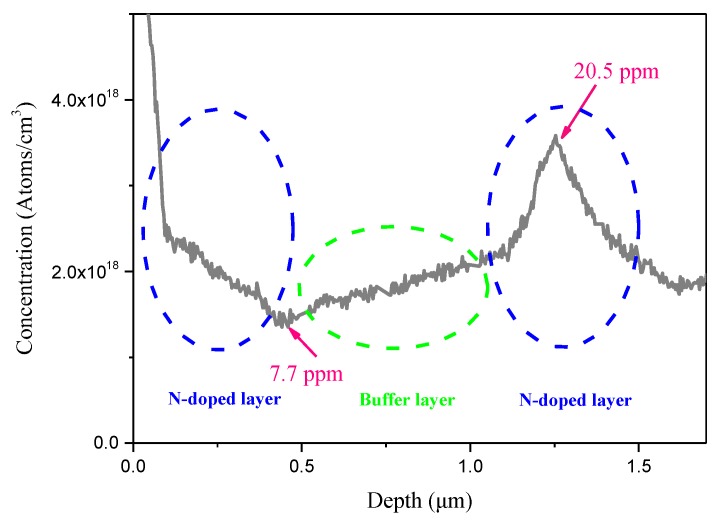
Secondary ion mass spectroscopy (SIMS) profile of the thin nitrogen-doped multilayers of sample 2.

We have studied the distribution of nitrogen in the thin nitrogen-doped multilayers by SIMS. The obtained SIMS profile (Figure 8) indicates the peak nitrogen concentration of ~20.5 ppm for the nitrogen-doped layer in the blue dotted line and the lowest nitrogen concentration of ~7.7 ppm for the buffer layer indicated by the green dotted line, respectively.

### 3.3. Mechanical Properties of the Multilayered Single Crystal Diamond

**Figure 9:**
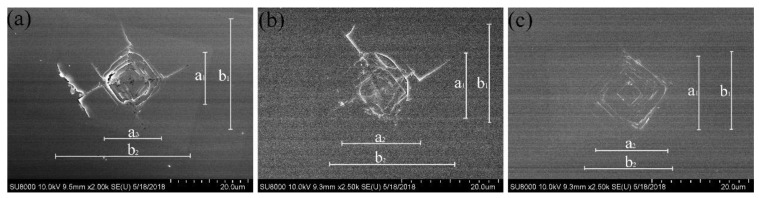
The indented surfaces of the samples viewed via SEM, in which the parameters a_1_, a_2_, b_1_, and b_2_ used to determine the Vickers hardness and fracture toughness are indicated. (**a**) The indented surface of the Ib-type HPHT seed substrate. (**b**,**c**) The indented surfaces of the samples 3 and 2, respectively.

Figure 9a,b show the indented surfaces of the Ib-type HPHT seed substrate and sample 3, respectively. Both samples show square crack patterns along the <110> direction and still exhibit cross-like <100> cracks. Figure 9c shows the indented morphology of the sample 2, which exhibits square cracks along the <110> direction and no cross-like cracks along <100>; the indentation produced by the pyramidal indenter clearly appears on the surface. Table 2 shows the Vickers hardness and fracture toughness on the {100} faces of the samples in the <100> direction. Hv and Kc were determined from Hv = 1854 P/a^2^, Kc = (0.016 ± 0.004) (E/Hv)^1/2^ (P/b^3/2^), where P (N) is the applied load, a (μm) is the arithmetic mean of the two diagonals of the Vickers indentation, b = (b_1_ + b_2_)/4, b_1_ and b_2_ are the lengths of the two diagonal cracks, and E is Young’s modulus (assumed to be 1000 GPa) [38,39]. The indentation measurements reveal that the sample 2 is extremely tough (22.0 ± 3.5 MPa m^1/2^), much tougher than the Ib-type HPHT seed substrate (8.3 ± 2.5 MPa m^1/2^) and the sample 3 (14.3 ± 3.0 MPa m^1/2^). Each sample was tested four times, and corresponding hardness and toughness were obtained by taking the average value.

These observations indicate that the nitrogen-doped multilayer CVD single crystal diamond is, in fact, tougher than the Ib-type HPHT seed substrate and the unintentionally doped CVD single crystal diamond.

## 4. Conclusions

Defects at the interface between the substrate and the epitaxial layer can induce dislocation, and more nitrogen-related defects are included into the pyramidal region. We have succeeded in fabricating thin nitrogen-doped multilayer single crystal diamond on (100)-oriented HPHT seed substrate under high power densities using MPCVD technology. When the thickness of the nitrogen-doped and the buffer layers was about 650 nm each, the fracture toughness of the thin nitrogen-doped multilayer CVD single crystal diamond was much higher than that of the HPHT seed substrate and the unintentionally doped CVD single crystal diamond. Further improvements will include the growth of thinner nitrogen-doped layers. The present study may provide the guidance for the future synthesis of ultra-tough and ultra-hard materials consisting of alternately-stacked and differently nitrogen-doped diamond layers. This method induces novel toughening and hardening of CVD single crystal diamonds.

## Figures and Tables

**Table 1:** Growth parameters for 3 different samples.

Samples	CH_4_/H_2_ (%)	H_2_ (sccm)	N_2_ (sccm)	Number of Layers	Total Thickness of the CVD Layer (μm)
#1	5	300	0.03/0.06/0.09/0.12/0.15	11	680
#2	5	300	0.09	55	35
#3	5	300	0	1	38

**Table 2:** Vickers hardness and fracture toughness of different samples.

Samples	A (μm)	B (μm)	Vickers Hardness (GPa)	Fracture Toughness (MPa m^1/2^)
Seed	14.1	15.8	91.0 ± 10	8.3 ± 2.5
2#	15.0	8.6	79.9 ± 14	22.0 ± 3.5
3#	15.1	11.49	79.8 ± 12	14.3 ± 3.0
